# Design and control of a self-adjusting outdoor landscape wall

**DOI:** 10.1038/s41598-023-42881-w

**Published:** 2023-09-20

**Authors:** Ru Cui

**Affiliations:** 1 School of Arts, Shaanxi University of Technology, Hanzhong, 723001 China; 2 UCAM Universidad Católica San Antonio de Murcia, Campus de los Jerónimos, No 135, 30107 Guadalupe, Murcia Spain

**Keywords:** Engineering, Physics

## Abstract

This paper designs an outdoor landscape wall, which is equipped with an electrical motor, a sunlight intensity sensor and a rain sensor. The plants in the landscape wall will be rotated indoors when the weather is bad. To the contrary, based on signals from the sunlight intensity sensor, the plants can be rotated outdoors to get suitable sunlight. The dynamics of the electrical motor current control loop is much higher than the requirement of the position control loop. The whole control system can be divided into two subsystems: out-loop position control system and inner-loop current control system. An adaptive control strategy is proposed for out-loop position control. A nonlinear controller based on feedback linearization is developed for inner-loop current control. The two subsystems are synthesized with a first-order filter. Simulations are conducted to verify the proposed control strategy. The simulation results demonstrate that high-performance position tracking can be achieved under parameter uncertainty and disturbance.

## Introduction

Outdoor landscape walls are widely used for decoration and air purification. However, current landscape walls do not have flexible doors, which makes the plans inside are not resistant to rain or snow. In addition, they cannot get suitable sunlight to help plants grow neither. Therefore, a self-adjusting outdoor landscape wall is designed, equipped with an electrical motor, a sunlight intensity sensor and a rain sensor. Thus, the plants can be rotated indoors according to the signals of the sensors.

High-performance control of electrical motors is still a great challenge. Proportional-integral-derivative (PID) control^1,2^ is a conventional method in electrical motor control. But PID controller is designed based on a certain working condition, and it cannot achieve best control performance in different working conditions. Feedback linearization^3–5^ can compensate the system nonlinear parts, which need an accurate system model. Adaptive control^6–8^ can improve the control performance under system uncertainties and disturbances through parameter adaption. However, adaptive control cannot handle disturbances or noises very well. Sliding mode control^9–11^ also can deal with system uncertainties and disturbances, but it may excite the system high frequency modes. Adaptive robust control (ARC)^12–15^ combined adaptive control and sliding mode control together. Disturbance observer^16–19^ is another effective approach to handle system disturbances, which considers the disturbances and system uncertainties as a whole and neglect the parameter uncertainties. Fuzzy logic^20–22^ and neural network^23,24^ based adaptive control strategies are also developed to achieve a better motion control performance. But adaptive controller based on fuzzy logic and neural network increases the complexity of the control system and the control parameters are hard to tune.

In this paper, a self-adjusting outdoor landscape wall is designed to keep plants in a better condition, which means the outdoor landscape wall can keep the plants in a suitable condition of sunlight or rain through adjusting the position of the plants. The designed outdoor landscape wall is driven by an electrical motor. The dynamic requirement of the motor position control is not very high and is much lower than the dynamics of the current control. Therefore, the whole system can be divided into two subsystems: out-loop position control system and inner-loop current control system. Adaptive control is employed for out-loop position control to compensate system uncertainties and disturbances. Feedback linearization is used in inner-loop current control to decide the actual control input. The controllers of out loop and inner loop are synthesized with a first-order filter. The input of the filter is the desired torque, while the output is the corresponding current and its derivative. This work makes the following contributions:

1. A new self-adjusting outdoor landscape wall is designed, which can keep the plants in a better condition.
2. According to system characteristic, the whole system is divided into two subsystems. A first-order filter is used to synthesize out-loop and inner-loop controllers. It avoids the differential explosion problem in conventional backstepping controller design procedure.

This article is organized as follow. The design of the self-adjusting outdoor landscape wall is showed in “Design of the self-adjusting outdoor landscape wall” section; the system dynamic model is established in “System dynamic model” section; the developed control strategy is presented in “Control strategy design” section; simulations are conducted in “Simulations” section; conclusions are drawn in “Conclusions” section.

## Results

### Design of the self-adjusting outdoor landscape wall

**Figure 1:**
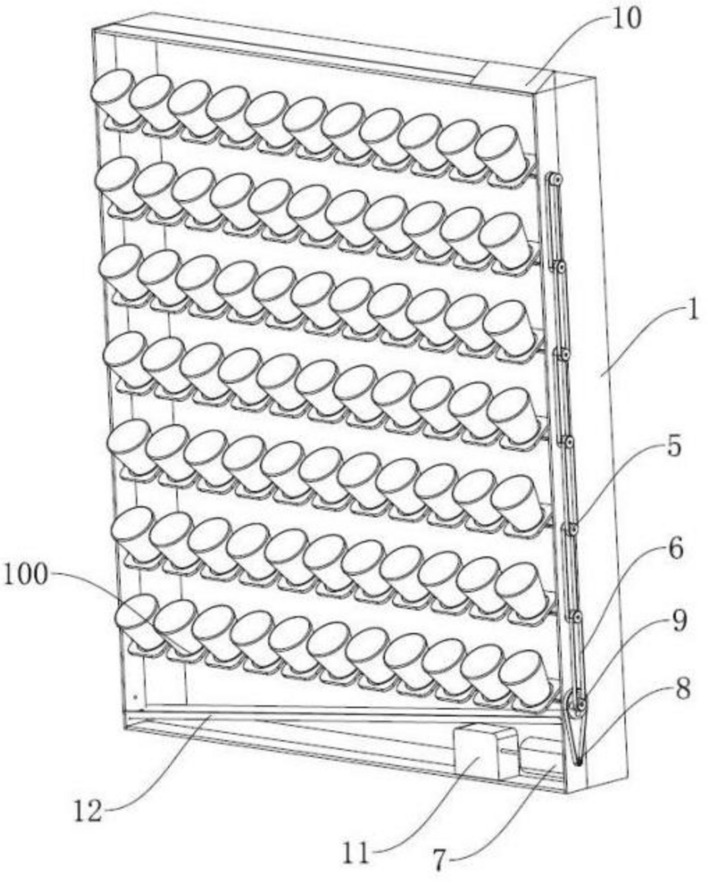
Structure of the designed self-adjusting outdoor landscape wall.

**Figure 2:**
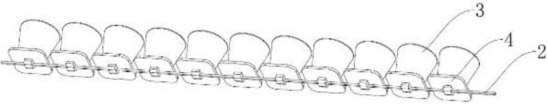
Structure of flowerpot modules 100.

A self-adjusting outdoor landscape wall is designed and shown in Fig. 1. Several rows of flowerpot modules 100 are arranged in frame 1 from top to bottom. As shown in Fig. 2, flowerpot modules 100 includes a rotating shaft 2 and flowerpots 3 fixed on rotating shaft 2. The rotating shaft 2 is arranged on both sides of frame 1, and its end extends out of one side of frame 1. Flowerpot mounting supports 4 are arranged on rotating shaft 2. A flowerpot 3 is fixed on each flowerpot mounting support 4. Flowerpot 3 and flowerpot mounting support 4 are connected by bolts or clamps. The bottom of flowerpot mounting support 4 has a shaped hole. And rotating shaft 2 passes through the shaped hole to drive flowerpot mounting support 4 to rotate. In addition, the distance between each row is determined by the height of the plant and the size of flowerpot 3. The bottom of frame 1 is installed with a tilting water guide tank 12, which can guide water to the outlet. This design meets the requirements of different plant number on the landscape wall. The adjustment is flexible and easy to operate. Different number of plants can be arranged on different rows.

Frame 1 is equipped with a drive module to rotate shaft 2, which consists transmission wheel 5, transmission belt 6 and motor module. Transmission wheel 5 is fixed on the end of rotating shaft 2, which extends out the side of frame 1. The adjacent transmission wheels 5 are connected with transmission belt 6. The motor module includes motor 7, whose output end is equipped with an output wheel 8. Output wheel 8 is connected with transmission shaft 2. Transmission belt 6 connects transmission wheels 5 on rotating shaft 2 to realize synchronous turning control. The end of bottom rotating shaft 2 extending out the side of frame 1 is installed with an input wheel 9. And input wheel 9 and output wheel 8 are connected by transmission belt 6. The radius ratio of output wheel 9 and input wheel 8 is 1:5, i.e., the deceleration ratio is 5:1. The transmission is adapted to different flowerpot layout layers, which can be convenient and flexible to assembly. A signal acquisition module 10 is arranged at the top of frame 1. Signal acquisition module 10 includes a sunlight intensity sensor and a rainfall sensor. The motor module also includes the control board 11, which controls the rotation of motor 7 according to the signals from the sunlight intensity sensor or the rainfall sensor, even remote control commands.

This design has following advantages:

1. Plants on the landscape wall can be rotated inside for protection in bad weather.
2. Sunlight requirement of plants can be satisfied through keeping plants in suitable position.
3. Self-adjustment can be realized according to the signals from the sunlight intensity sensor or the rainfall sensor, even remote control commands.

### System dynamic model

The system dynamic model of the designed self-adjusting outdoor landscape wall can be simplified into

1 $$J\ddot{\theta }+B\dot{\theta }+d=T,$$

where $J$ is the is the lumped moment of inertia, $\theta$ is the rotational angle, $B$ is the viscous friction coefficient, $d$ represents the unmodeled forces and disturbances, and $T$ is the drive torque of the motor.

The drive torque of the motor can be expressed as

2 $$T={C}_{t}I,$$

where ${C}_{t}$ is the torque constant and $I$ is the current of the motor.

For the internal dynamics of the motor circuit,

3 $$RI+L\frac{dI}{dt}+{E}_{a}=U,$$

where $R$ is the resistance, $L$ is the inductance, $U$ is the input voltage. And ${E}_{a}$ is the back electromotive force, and ${E}_{a}={C}_{e}\dot{\theta }$, ${C}_{e}$ is the back electromotive force constant.

Let $x={\left[{x}_{1},{x}_{2},{x}_{3}\right]}^{T}={\left[\theta ,\dot{\theta },I\right]}^{T}$ be the state variables, the dynamics of the system can be rewritten in state space,

4 $$\begin{array}{*{20}l} {J\dot{x}_{2} + Bx_{2} + d = T} \hfill \\ {Rx_{3} + L\dot{x}_{3} + C_{e} x_{2} = U} \hfill \\ \end{array} .$$

### Control strategy design

The plants in the landscape wall can be rotated according to the signals from the sunlight intensity sensor or the rainfall sensor, even remote control commands. To make the plants in the landscape wall in a better condition, a controller is designed for the landscape wall under parameter uncertainties and disturbances. Adaptive control is employed for out-loop position control to compensate the system uncertainties and disturbances. Feedback linearization is used in inner-loop current control to decide the actual control input. The controllers of out loop and inner loop are synthesized with a first-order filter.

#### Out-loop position control

Define the position tracking error as ${z}_{1}={x}_{1d}-{x}_{1}$, a filtered tracking error ${z}_{2}={\dot{z}}_{1}+{k}_{1}{z}_{1}$, and an auxiliary signal $v={\dot{x}}_{1d}+{k}_{1}{z}_{1}$. ${x}_{1d}$ and ${\dot{x}}_{1d}$ are the desired positon and its derivative. ${k}_{1}$ is a positive constant. And let $z={\left[{z}_{1},{z}_{2}\right]}^{T}$.

From (4), it can be obtained

5 $$J\dot{z}_{2} = J\left( {\ddot{z}_{1} + k_{1} \dot{z}_{1} } \right) = \underbrace {{J\dot{v} + Bv + d}}_{H} - Bz_{2} - T = H - Bz_{2} - T,$$

where $H$ is a lumped uncertainty, and is dependent on $J$, $B$, $d$.

The approximation of $H$ can be expressed in the forms of approximations $\widehat{J}$, $\widehat{B}$ and $\widehat{d}$,

6 $$\widehat{H}=\widehat{J}\dot{v}+\widehat{B}v+\widehat{d}.$$

The approximation error of $H$ is defined as

7 $$\widetilde{H}=H-\widehat{H}=\widetilde{J}\dot{v}+\widetilde{B}v+\widetilde{d},$$

where $$\widetilde{J}=J-\widehat{J}$$, $$\widetilde{B}=B-\widehat{B}$$, and $$\widetilde{d}=d-\widehat{d}$$.

In a compact expression, let $H={\Phi }^{T}W$ and $\widehat{H}={\Phi }^{T}\widehat{W}$, where $\Phi ={\left[\dot{v},v,1\right]}^{T}$, $W={\left[J,B,d\right]}^{T}$, $\widehat{W}={\left[{\widehat{W}}_{1},{\widehat{W}}_{2},{\widehat{W}}_{3}\right]}^{T}={\left[\widehat{J},\widehat{B},\widehat{d}\right]}^{T}$. And define $\widetilde{W}=W-\widehat{W}$.

The control input torque is designed as

8 $${T}_{d}=Kz+\widehat{H},$$

where $K=\left[1,{k}_{2}\right]$, ${k}_{2}$ is positive constant.

And the adaptive law is designed as

9 $${\dot{\widehat{W}}}_{i}=Pro{j}_{{\widehat{W}}_{i}}\left({\Gamma }_{i}\Phi {z}_{2}\right),$$

where $\Gamma =diag\left({\Gamma }_{1},{\Gamma }_{2},{\Gamma }_{3}\right)$ is a positive definite diagonal matrix, $i=\mathrm{1,2},3$. And $Pro{j}_{{\widehat{W}}_{i}}\left(\bullet \right)$ is a projection function

10 $$Proj_{{\hat{W}_{i} }} \left( \cdot \right) = \left\{ {\begin{array}{*{20}c} 0 & {{\text{if }}\left\{ {\begin{array}{*{20}c} {\hat{W}_{i} = \hat{W}_{i\max } \& \cdot > 0} \\ {\hat{W}_{i} = \hat{W}_{i\min } \& \cdot < 0} \\ \end{array} } \right.} \\ \cdot & {{\text{otherwise}}} \\ \end{array} } \right.,$$

where ${\widehat{W}}_{imax}$ and ${\widehat{W}}_{imin}$ are known, $i=\mathrm{1,2},3$.

Define a following Lyapunov function

11 $${V}_{1}=\frac{1}{2}{z}_{1}^{2}+\frac{1}{2}J{z}_{2}^{2}+\frac{1}{2}\widetilde{W}{\Gamma }^{-1}{\widetilde{W}}^{2}.$$

The time derivative of ${V}_{1}$ is given by

12 $${\dot{V}}_{1}={z}_{1}{\dot{z}}_{1}+J{z}_{2}{\dot{z}}_{2}+\widetilde{W}{\Gamma }^{-1}\dot{\widetilde{W}}={z}_{1}\left({z}_{2}-{k}_{1}{z}_{1}\right)+{z}_{2}\left(H-B{z}_{2}-T\right)-\widetilde{W}{\Gamma }^{-1}\dot{\widehat{W}}\le -{k}_{1}{z}_{1}^{2}-{k}_{2}{z}_{2}^{2}-B{z}_{2}^{2}.$$

Because viscous friction coefficient of practice system $B\ge 0$, ${\dot{V}}_{1}\le 0$. Thus, the stability of the out-loop position control can be guaranteed.

#### Inner-loop current control

Define the position Let ${x}_{3d}$ be the desired current. And define ${z}_{3}={x}_{3d}-{x}_{3}$. So,

13 $${\dot{z}}_{3}={\dot{x}}_{3d}-{\dot{x}}_{3}={\dot{x}}_{3d}-\frac{1}{L}\left(U-R{x}_{3}-{C}_{e}{x}_{2}\right).$$

Define the control input voltage as

14 $$U=R{x}_{3}+{C}_{e}{x}_{2}+L\left({\dot{x}}_{3d}+{k}_{3}{z}_{3}\right),$$

where ${k}_{3}$ is a positive constant.

Define a following Lyapunov function

15 $${V}_{2}=\frac{1}{2}{z}_{3}^{2}.$$

The time derivative of ${V}_{2}$ is given by

16 $${\dot{V}}_{2}={z}_{3}{\dot{z}}_{3}={z}_{3}\left[{\dot{x}}_{3d}-\frac{1}{L}\left(U-R{x}_{3}-{C}_{e}{x}_{2}\right)\right]=-{k}_{3}{z}_{3}^{2}.$$

Therefore, ${\dot{V}}_{2}\le 0$, the stability of the inner-loop current control can be guaranteed.

#### First-order filter

**Figure 3:**
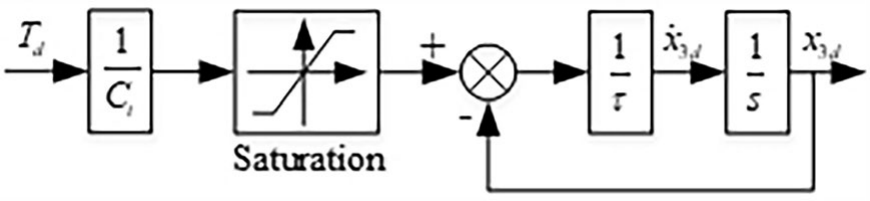
Schematic diagram of the first-order filter.

The controllers of out loop and inner loop are synthesized with a first-order filter to avoid the differential explosion problem in conventional backstepping controller design procedure. Figure 3 shows the first-order filter. The input of the filter is the out-loop desired torque ${T}_{d}$, and the output is the inner-loop desired current ${x}_{3d}$ and its derivative ${\dot{x}}_{3d}$. The saturation function keeps the desired current ${x}_{3d}$ in an appropriate range. Besides, the time constant $\tau$ should be chosen according to the properties of the selected motor.

**Figure 4:**
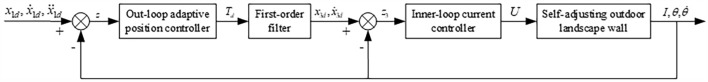
Schematic diagram of the proposed adaptive controller.

The stabilities of inner loop and out loop are guaranteed. And the dynamic requirement of the out-loop position control is not very high and is much lower than the dynamics of the inner-loop current control. The two controllers are synthesized with a first-order filter. Therefore, the stability of the whole system can be guaranteed. The proposed adaptive controller for the designed self-adjusting outdoor landscape wall is shown in Fig. 4.

### Simulations

Numerical simulations are conducted to verify the proposed adaptive controller (AC) for the designed outdoor landscape wall. The parameters of the motor and system are shown in Table 1. Four controllers are compared. The first is PID, i.e., $U={k}_{P}{z}_{1}+{k}_{I}\int {z}_{1}dt+{k}_{D}\frac{d{z}_{1}}{dt}$, whose parameters are shown in Table 2. And the parameters cannot set bigger, because oscillation will occur. The second is nonlinear controller without parameter adaption (NC), i.e., $\Gamma =0$, the rest parameters are same as AC. The third is sliding mode controller (SMC). SMC is based on NC, and its control input torque is designed as ${T}_{d}= Kz+\widehat{H}+{k}_{3}sgn\left({z}_{2}\right), {k}_{3}=0.2$. The third is AC, whose parameters are shown in Table 3.

**Table 1:** Parameters of the motor and system.

Parameter	Value
$J$	0.028 ${\text{kg}}{ \, {\text{m}}}^{2}$
$B$	0.01 $\frac{\text{Nm}}{\left(\frac{\text{rad}}{s}\right)}$
${C}_{t}$	0.231 $\frac{\text{Nm}}{A}$
${C}_{e}$	0.23 $\frac{V}{\left(\frac{\text{rad}}{s}\right)}$
$R$	0.854 $\Omega$
$L$	1.07 ${\text{mH}}$

**Table 2:** Parameters of PID.

Parameter	Value
${k}_{P}$	1200
${k}_{I}$	100
${k}_{D}$	20

**Table 3:** Parameters of AC and SMC.

Parameter	Value
${k}_{1}$	10
${k}_{2}$	10
$\Gamma$	diag(1,1,1)
$\tau$	0.01s

**Figure 5:**
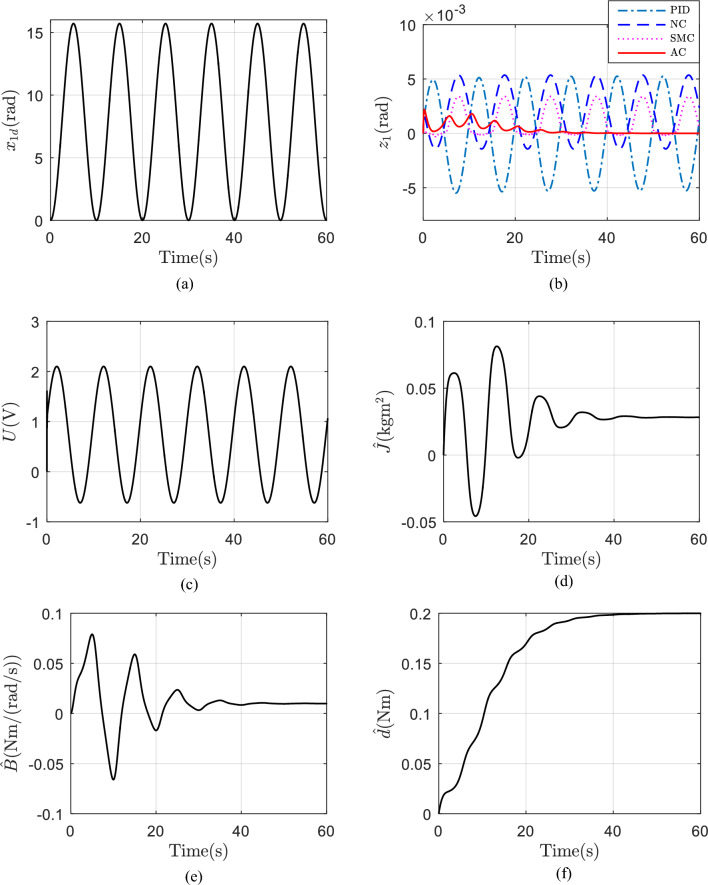
(**a**) Desired trajectory; (**b**) tracking errors with a constant d; (**c**) control voltage of AC with a constant d; (**d**) estimation of J with a constant d; (**e**) estimation of B with a constant d; (**f**) estimation of d with a constant d.

The quantity and scale of the plants in the landscape wall can be various, that means J, B are unknown. The tested desired trajectory is shown in Fig. 5a. Firstly, assuming d is constant, i.e., d = 0.2 Nm. The initial values of the adaptive parameters in AC are set as 0. The values of J, B of NC are set as the 0.8 times of the true values, so does SMC. The tracking errors of the four controllers are shown in Fig. 5b. Among the four controllers, AC demonstrates the smallest errors and the error will converge to zero finally. SMC obtain better control performance comparing with NC, because sliding mode term is applied. The control voltage of AC is shown in Fig. 5c. The estimated parameters of AC are shown in Fig. 5d–f. The estimations converge to their true values gradually.

**Figure 6:**
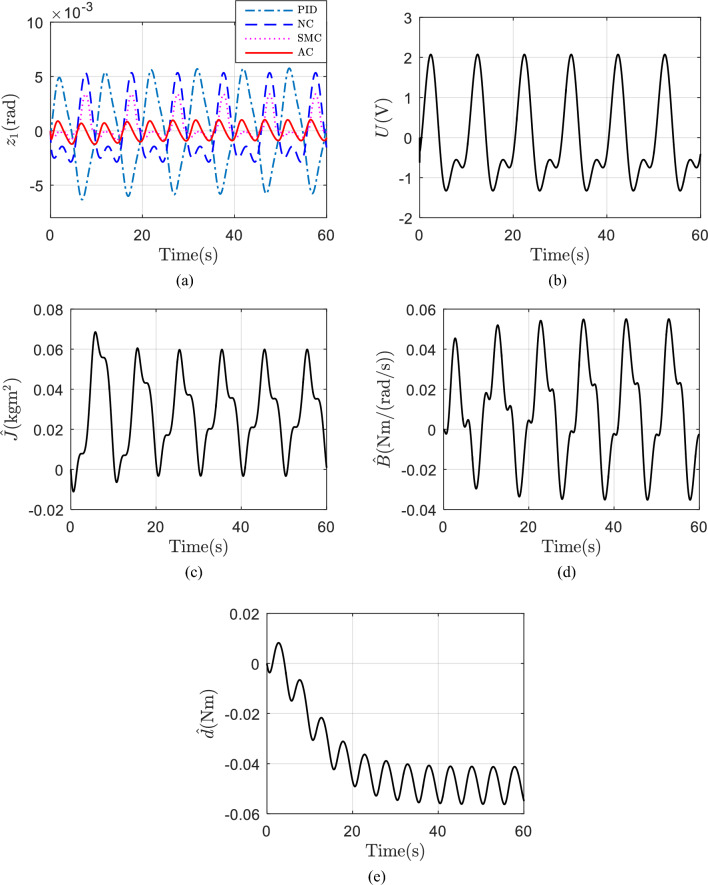
(**a**) Tracking errors with a variable d; (**b**) control voltage of AC with a variable d; (**c**) estimation of J with a variable d; (**d**) estimation of B with a variable d; (**e**) estimation of d with a variable d.

Secondly, assuming $d$ is variable, i.e., $d=0.2{\text{ sin}}\left(\frac{\pi }{10}t\right){\text{ Nm}}$. Same desired trajectory shown in Fig. 5a are tested. The initial values of the adaptive parameters in AC and parameters in NC and SMC are set the same as before. Figure 6a shows the tracking errors of the four controllers. AC still has the best tracking performance. The control voltage of AC is shown in Fig. 6b. The estimated parameters of AC are shown in Fig. 6c–e. The reason why the tracking errors of AC does not converge to zero and its parameter estimations cannot converge to true values is that a variable $d$ is used in simulation. But even with a variable $d$, AC still shows the best tracking performance, which demonstrates the universality of AC.

**Figure 7:**
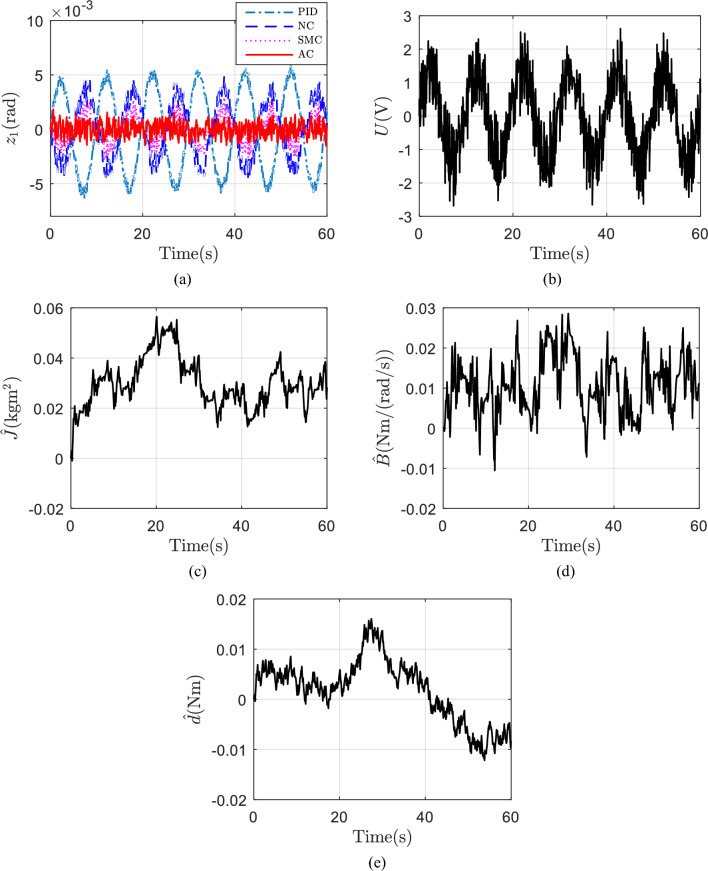
(**a**) Tracking errors with a random d; (**b**) control voltage of AC with a random d; (**c**) estimation of J with a random d; (**d**) estimation of B with a random d; (**e**) estimation of d with a random d.

Finally, assuming $d$ is random, i.e., uniform random number of Matlab is used, and its maximum and minimum value is $\pm \,0.2{\text{ Nm}}$ and sample time is 0.1 s. Same desired trajectory shown in Fig. 5a are tested. The initial values of the adaptive parameters in AC and parameters in NC are set the same as before. Figure 7a shows the tracking errors of the four controllers. AC still has the best tracking performance. The control voltage of AC is shown in Fig. 7b. The estimated parameters of AC are shown in Fig. 7c–e. Why the tracking errors of AC does not converge to zero and why its parameter estimations cannot converge to true values, the reason is same. But even with a random $d$, AC still shows the best tracking performance, which also demonstrates the universality of AC.

## Discussion

In this paper, a new self-adjusting outdoor landscape wall is designed. The outdoor landscape wall is driven by an electrical motor and equipped with a sunlight intensity sensor and a rain sensor. The plants in the landscape wall can be rotated according to the signals from the sensors. The dynamics of the electrical motor current control loop is much higher than the requirement of the position control loop. The whole control system can be divided into two subsystems: out-loop position control system and inner-loop current control system. A position controller based on adaptive control are proposed for out loop to deal with different kinds and arrangements of plants. A nonlinear current controller based on feedback linearization is developed for inner loop. The two controllers are synthesized with a first-order filter. Simulations with different d are conducted to verify the proposed AC strategy. The simulation results demonstrate that high-performance position tracking can be achieved under parameter uncertainty and disturbance.

## Data Availability

The datasets used and analysed during the current study available from the corresponding author Ru Cui on reasonable request.
